# RCSB Protein Data Bank: improved annotation, search and visualization of membrane protein structures archived in the PDB

**DOI:** 10.1093/bioinformatics/btab813

**Published:** 2021-12-02

**Authors:** Sebastian Bittrich, Yana Rose, Joan Segura, Robert Lowe, John D Westbrook, Jose M Duarte, Stephen K Burley

**Affiliations:** Research Collaboratory for Structural Bioinformatics Protein Data Bank, San Diego Supercomputer Center, University of California, La Jolla, CA 92093, USA; Research Collaboratory for Structural Bioinformatics Protein Data Bank, San Diego Supercomputer Center, University of California, La Jolla, CA 92093, USA; Research Collaboratory for Structural Bioinformatics Protein Data Bank, San Diego Supercomputer Center, University of California, La Jolla, CA 92093, USA; Research Collaboratory for Structural Bioinformatics Protein Data Bank, Rutgers, The State University of New Jersey, Piscataway, NJ 08854, USA; Institute for Quantitative Biomedicine, Rutgers, The State University of New Jersey, Piscataway, NJ 08854, USA; Research Collaboratory for Structural Bioinformatics Protein Data Bank, Rutgers, The State University of New Jersey, Piscataway, NJ 08854, USA; Institute for Quantitative Biomedicine, Rutgers, The State University of New Jersey, Piscataway, NJ 08854, USA; Cancer Institute of New Jersey, Rutgers, The State University of New Jersey, New Brunswick, NJ 08901, USA; Research Collaboratory for Structural Bioinformatics Protein Data Bank, San Diego Supercomputer Center, University of California, La Jolla, CA 92093, USA; Research Collaboratory for Structural Bioinformatics Protein Data Bank, San Diego Supercomputer Center, University of California, La Jolla, CA 92093, USA; Research Collaboratory for Structural Bioinformatics Protein Data Bank, Rutgers, The State University of New Jersey, Piscataway, NJ 08854, USA; Institute for Quantitative Biomedicine, Rutgers, The State University of New Jersey, Piscataway, NJ 08854, USA; Cancer Institute of New Jersey, Rutgers, The State University of New Jersey, New Brunswick, NJ 08901, USA; Department of Chemistry and Chemical Biology, Rutgers, The State University of New Jersey, Piscataway, NJ 08854, USA

## Abstract

**Motivation:**

Membrane proteins are encoded by approximately one fifth of human genes but account for more than half of all US FDA approved drug targets. Thanks to new technological advances, the number of membrane proteins archived in the PDB is growing rapidly. However, automatic identification of membrane proteins or inference of membrane location is not a trivial task.

**Results:**

We present recent improvements to the RCSB Protein Data Bank web portal (RCSB PDB, rcsb.org) that provide a wealth of new membrane protein annotations integrated from four external resources: OPM, PDBTM, MemProtMD and mpstruc. We have substantially enhanced the presentation of data on membrane proteins. The number of membrane proteins with annotations available on rcsb.org was increased by ∼80%. Users can search for these annotations, explore corresponding tree hierarchies, display membrane segments at the 1D amino acid sequence level, and visualize the predicted location of the membrane layer in 3D.

**Availability and implementation:**

Annotations, search, tree data and visualization are available at our rcsb.org web portal. Membrane visualization is supported by the open-source Mol* viewer (molstar.org and github.com/molstar/molstar).

**Supplementary information:**

Supplementary data are available at *Bioinformatics* online.

## 1 Introduction

Membranes define cellular and organellar boundaries. They are composed of phospholipid bilayers. Membrane proteins are either embedded in or associated with the phospholipid bilayer. Membrane proteins are crucial for cell survival and communication across membranes, serving as molecular transporters, signal receptors, ion channels and even enzymes. Recent improvements in experimental methods (e.g. use of cryo-electron microscopy and inclusion of detergents, lipid molecules, vesicles and nanodiscs) are providing a wealth of new possibilities for membrane protein structure determination

Membrane proteins have diverse spatiotemporal characteristics. Integral membrane proteins are permanently attached to a lipid bilayer while peripheral ones form transient complexes with the membrane. Transmembrane proteins traverse the membrane bilayer at least once, whereas monotopic membrane proteins are attached to a single face of the lipid bilayer. Information on membrane proteins provided by dedicated resources such as OPM (Lomize *et al.*, 2012), PDBTM (Kozma *et al.*, 2013), MemProtMD (Newport *et al.*, 2019) and mpstruc (White, 2009) differ in coverage (Shimizu *et al.*, 2018) and type of available information (see Supplementary Tables S1 and S2).

Historically, this complexity made it challenging for users to explore the plethora of information on membrane proteins freely available from the Protein Data Bank (PDB) archive. Herein, we present new features that provide consistent ways to search, browse and visualize membrane proteins by integrating information from trusted external sources into the recently streamlined RCSB PDB data management and delivery architecture (Burley *et al.*, 2021; Rose *et al.*, 2021), emphasizing flexibility, fidelity, maintainability and sustainability.

## 2 Results

On June 16 2021, the PDB archive housed 10 133 polymer entities annotated as membrane proteins by the previously integrated mpstruc resource. The newly integrated trusted resources (OPM, PDBTM and MemProtMD) increased coverage by ∼80% to 18 247 (see Supplementary Table S2). On the rcsb.org web portal, users can search, browse and visualize data on membrane proteins independent of annotation provenance. Links to the external data resources provide details, such as protein classification, amino acid sequence-level data or curated membrane locations (see Supplementary Table S1). To aid PDB data consumers in analyzing their search results, we display the distribution of hits annotated as membrane proteins in the search result Refinements panel (see Supplementary Fig. S1). Clicking on a membrane resource will drill-down into a subset of the results. Membrane annotations are programmatically accessible *via* RCSB PDB Search (search.rcsb.org), Data (data.rcsb.org) and Annotation APIs (1d-coordinates.rcsb.org).

### 2.1 Improved structure summary page

**Fig. 1.**
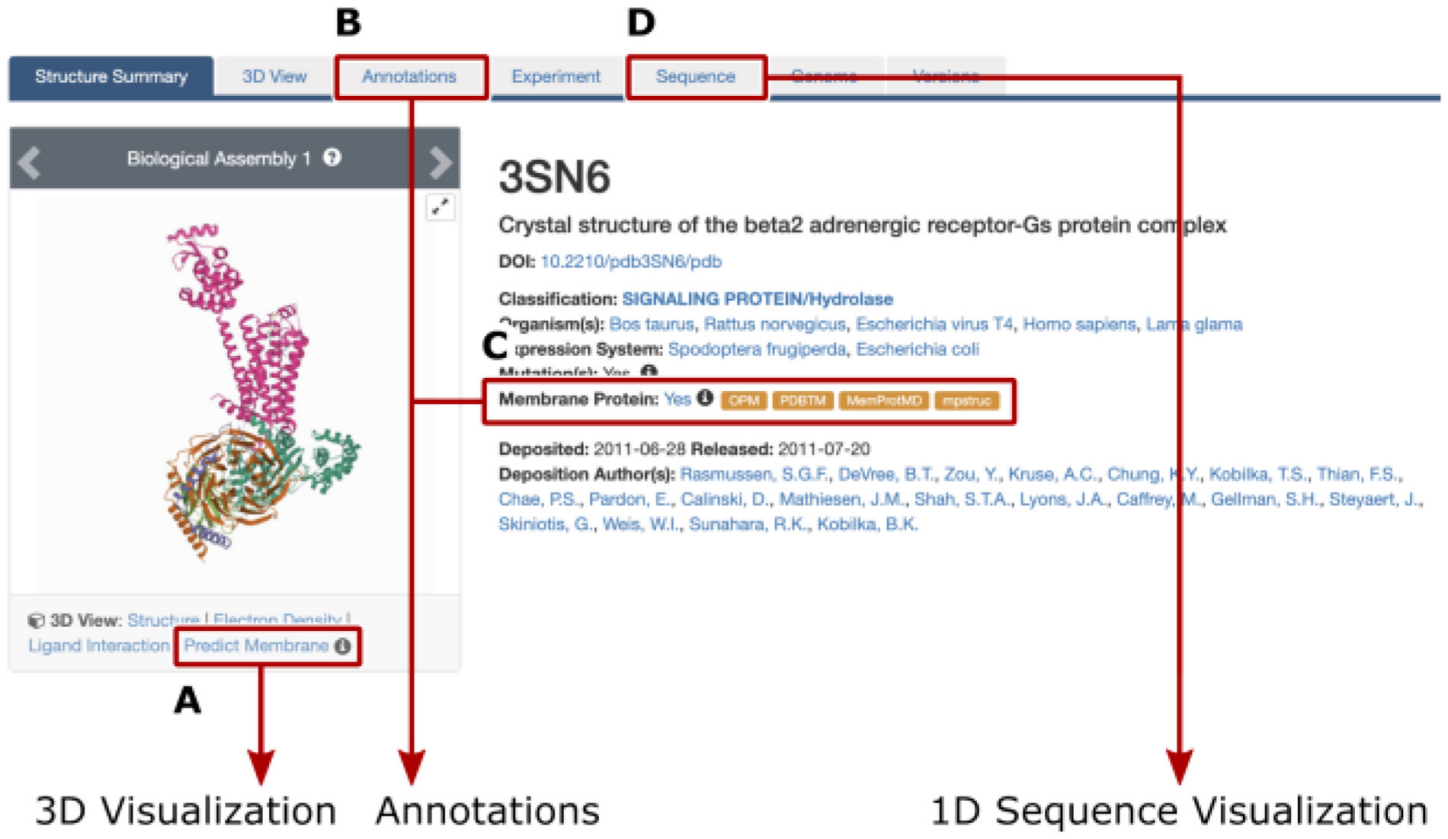
Tabs in the header of the RCSB PDB structure summary page provide an overview of available information on membrane proteins. (**A**) Visualization of predicted membrane orientation in Mol*. (**B**) Annotation details. (**C**) Orange boxes provide access to integrated external resources. (**D**) 1D visualization of membrane segments in Protein Feature View

We have revamped the RCSB PDB Structure Summary page (Fig.  1), which provides summary information for each PDB entry. PDB entries are designated with a four-character alphanumeric PDB ID (e.g. 3SN6) and contain at least one polymer entity, which refer to chemically unique molecules in an entry. Detailed definitions can be found in (Burley *et al.*, 2021).

Annotations integrated from external resources and links enable users to access additional details. Entities are annotated as membrane proteins if applicable. The entire PDB structure is annotated as membrane protein (Fig.  1C) if at least one entity is annotated as either transmembrane or membrane-associated by OPM, PDBTM, MemProtMD or mpstruc. The ‘Membrane Protein’ link in blue font (Fig.  1C) takes users to the Annotation tab of the structure entry (see Supplementary Fig. S3). With the exception of mpstruc, links in the orange boxes lead to structure-specific pages of the integrated external resources.

### 2.2 Visualize predicted membrane location in Mol*

We contributed a new implementation of the ANVIL algorithm (Postic *et al.*, 2016) to the Mol* package. The algorithm uses only 3D structure information to predict the membrane location. ANVIL is a simplified version of the TMDET algorithm (Tusnády *et al.*, 2004) used by PDBTM. The Mol* 3D viewer (Sehnal *et al.*, 2021) was extended with a customized set of membrane visualization tools (see Supplementary Fig. S2) that display predicted membrane boundaries. (N.B.: This visualization is independent of annotation provenance.) The RCSB image gallery allows access to this visualization for specific assemblies or the crystallographic asymmetric unit (Fig.  1A). Users should always visit external resources for reliable membrane location predictions. The ANVIL implementation is merely a visualization tool and may output flawed predictions (see Supplementary Table S3 for examples).

### 2.3 Membrane protein annotations

The Annotations page of each membrane protein structure contains a summary of extant annotations (see Supplementary Fig. S3). OPM and mpstruc provide detailed hierarchies, generic annotations are displayed for PDBTM and MemProtMD. Clicking a link highlighted with bold font will launch a search for polymer entities that share this annotation. All annotations are updated once per week.

### 2.4 Browse membrane annotation trees

Users can browse tree hierarchies provided by OPM and mpstruc using the Browse Annotations feature on the rcsb.org web portal. Increasingly fine-grained classifications are available by clicking on branches of the tree (see Supplementary Fig. S4). The link at the end of each line triggers the corresponding search and returns all matching entities. Like all annotation trees depicted on rcsb.org, the mpstruc and OPM tree can either be explored individually or accessed *via* the Advanced Search panel.

### 2.5 Explore membrane segments in the Protein Feature View

The OPM and PDBTM resources provide sequence-level data on segments that are embedded in or associated with a membrane. This information can be visualized in the Protein Feature View (Segura *et al.*, 2021), which allows exploring the relation of membrane segments to other 1D sequence features such as secondary structure elements or ligand binding sites, or 3D structure features (see Supplementary Fig. S5).

## 3 Conclusions

We report integration of information from four trusted membrane protein data resources. Coverage of membrane proteins in the rcsb.org web portal improved substantially and users now have access to new 1D and 3D visualizations for membrane proteins. Recent RCSB PDB led innovations (Rose *et al.*, 2021; Segura *et al.*, 2021) and the Mol* 3D viewer (Sehnal *et al.*, 2021), collaboratively developed by the Protein Data Bank in Europe and RCSB PDB, enabled seamless integration of new features into the rcsb.org web portal search infrastructure.

## Supplementary Material

btab813_Supplementary_Data
