# Supplementary material for: RCSB Protein Data Bank: improved annotation, search and visualization of membrane protein structures archived in the PDB
Source: Bioinformatics. 2021 Dec 2;38(5):1452–4. doi: 10.1093/bioinformatics/btab813 (PMC8826025; doi:10.1093/bioinformatics/btab813)
Supplement: btab813_Supplementary_Data [file btab813_supplementary_data.pdf]

# Supplementary Information

## Supplementary Table 1

| **Information Provided** | **OPM** | **PDBTM** | **MemProtMD** | **mpstruc** |
|---|---|---|---|---|
| Detailed Classification of Annotated Membrane Proteins | Integrated into rcsb.org as Browse Annotations Tree | N/A | N/A | Integrated into rcsb.org as Browse Annotations Tree |
| Membrane-Associated Sequence Segments | Integrated into rcsb.org as entity-level information | Integrated into rcsb.org as entity-level information | N/A | N/A |
| 3D Visualization of Membrane Layer | Available in external resource | Available in external resource | Available in external resource | N/A |

Resources differ in how they detect membrane proteins. OPM (Lomize *et al.*, 2012) uses SCOP and the Transporter Classification Database (TCDB) to annotate proteins and then employs a custom transfer energy function to determine the location of the membrane layer. PDBTM (Kozma *et al.*, 2012) screens for membrane proteins by the related TMDET algorithm (Tusnády *et al.*, 2004). MemProtMD (Newport *et al.*, 2019) employs an automatic pipeline that identifies integral alpha-helical domains and beta barrels based on sequence features. mpstruc (White, 2009) relies on the manual curation of literature.

This leads to differences regarding the covered types of membrane proteins. *E.g.*, OPM covers substantially more peripheral membrane proteins than other resources. In contrast, MemProtMD was specifically designed for integral membrane proteins.

Data from external resources is loaded as part of the RCSB weekly update workflow. Registered data files from each external resource are downloaded as part of this process. If a resource is temporarily unavailable the process can run with data that was obtained in the previous week. The RCSB weekly update workflow is flexible in the sense that it can ignore/drop external resources entirely in case of longer term disruptions to external data sources.

## Supplementary Table 2

| | OPM | PDBTM | MemProtMD | mpstruc | Total |
|---|---|---|---|---|---|
| # Entries | 5,042 | 5,695 | 5,158 | 4,339 | 8,337 |
| # Entities | 10,482 | 13,364 | 10,191 | 10,133 | 18,247 |

## Supplementary Table 3

The ANVIL algorithm outputs flawed predictions in some cases. Predictions on NMR models tend to be of low quality. Below are examples of problematic predictions.
If available, users should always visit OPM, PDBTM, and/or MemProtMD for more reliable predictions on the membrane location. We include ANVIL as a convenient visualization tool which is applicable for all annotated membrane entries, independent of provenance. Additionally, programmatic access to membrane sequence segments (but not membrane location) is provided at 1d-coordinates.rcsb.org, which delivers data from OPM and/or PDBTM.

| PDB ID | Problem |
|---|---|
| 1A0S | |
| 1AFO | NMR structure |
| 1ZZA | NMR structure |
| 4TKR | 2 orthogonally oriented chains in assembly |
| 4UWA | Large sections of unknown amino acids |
| 5X29 | NMR structure |
| 6B73 | |
| 7RNN | |

# Supplementary Figure 1

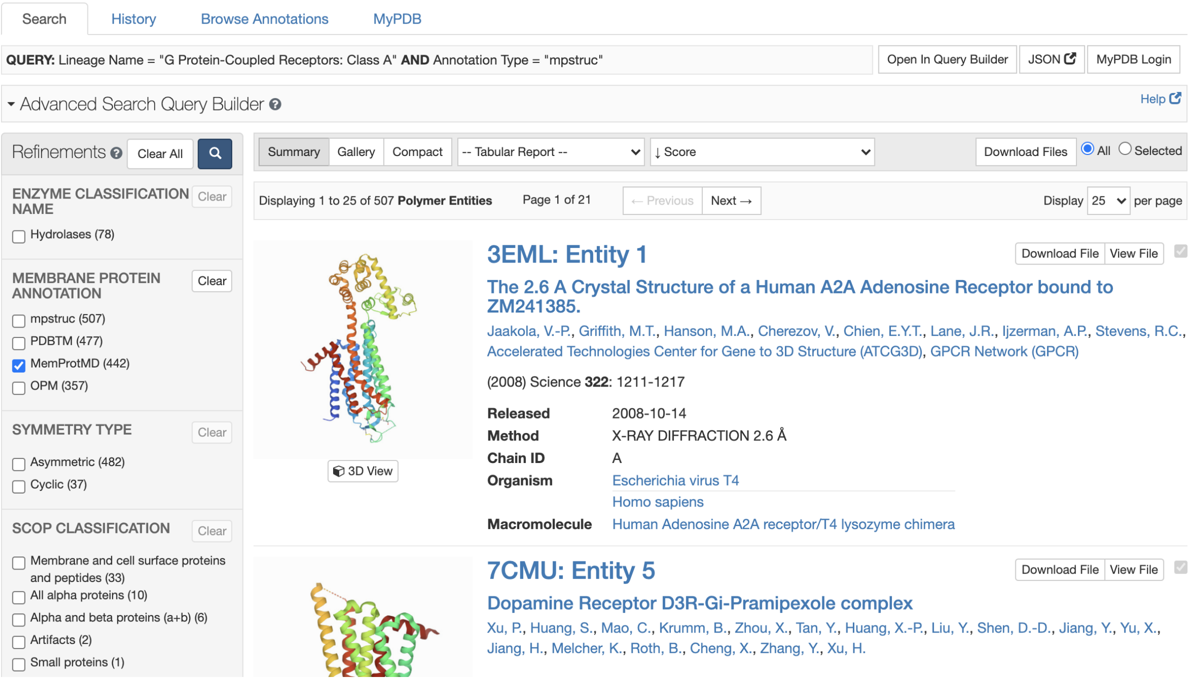


The 'Membrane Protein Annotation' drill-down menu on the left allows to refine searches, *e.g.* by requiring the presence of MemProtMD annotations.

# Supplementary Figure 2

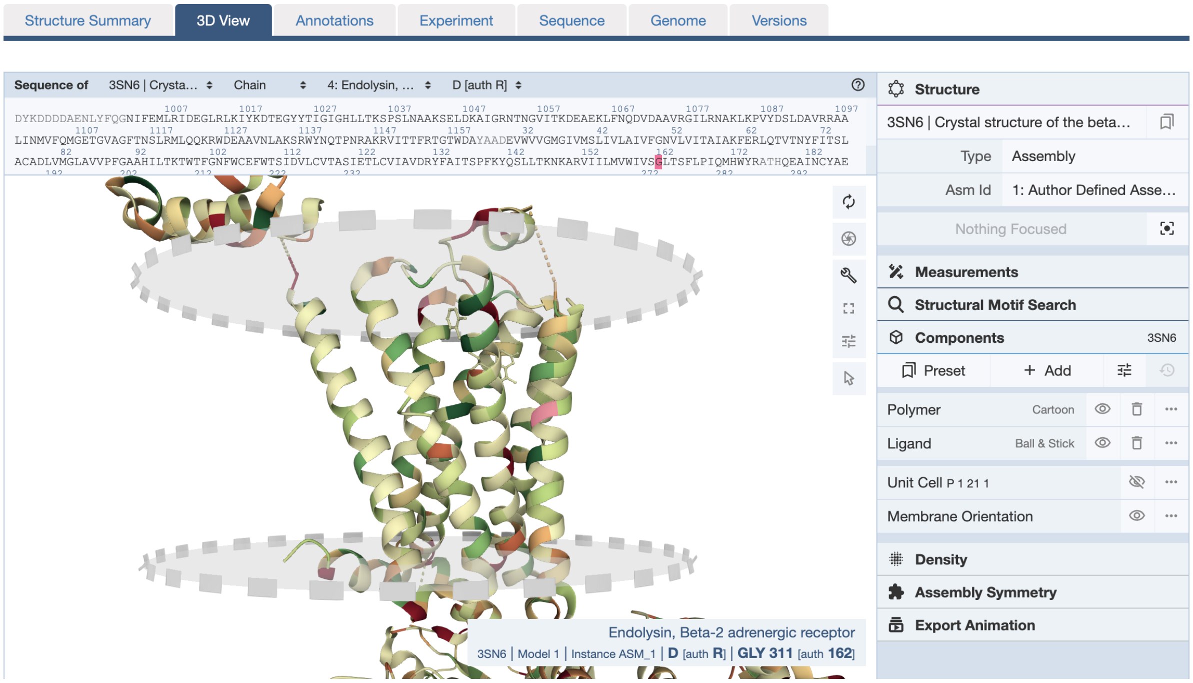


Predicted membrane position is shown by two translucent circular planes. Amino acids are colored by hydrophobicity.

# Supplementary Figure 3

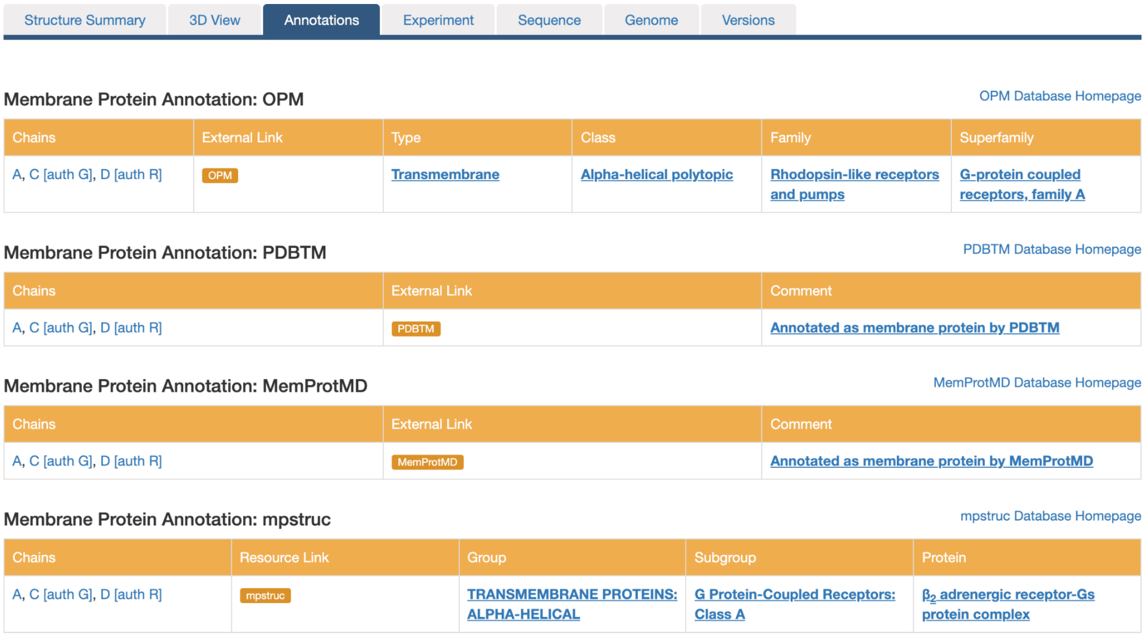


Membrane Protein Annotation: OPM

OPM Database Homepage

| Chains | External Link | Type | Class | Family | Superfamily |
|---|---|---|---|---|---|
| A, C [auth G], D [auth R] | OPM | Transmembrane | Alpha-helical polytopic | Rhodopsin-like receptors and pumps | G-protein coupled receptors, family A |

Membrane Protein Annotation: PDBTM

PDBTM Database Homepage

| Chains | External Link | Comment |
|---|---|---|
| A, C [auth G], D [auth R] | PDBTM | Annotated as membrane protein by PDBTM |

Membrane Protein Annotation: MemProtMD

MemProtMD Database Homepage

| Chains | External Link | Comment |
|---|---|---|
| A, C [auth G], D [auth R] | MemProtMD | Annotated as membrane protein by MemProtMD |

Membrane Protein Annotation: mpstruc

mpstruc Database Homepage

| Chains | Resource Link | Group | Subgroup | Protein |
|---|---|---|---|---|
| A, C [auth G], D [auth R] | mpstruc | TRANSMEMBRANE PROTEINS: ALPHA-HELICAL | G Protein-Coupled Receptors: Class A | $\beta_2$ adrenergic receptor-Gs protein complex |

Membrane protein annotations for PDB ID 3SN6. Orange boxes link to external resources. Bold links launch queries for entities that share these annotations.

# Supplementary Figure 4

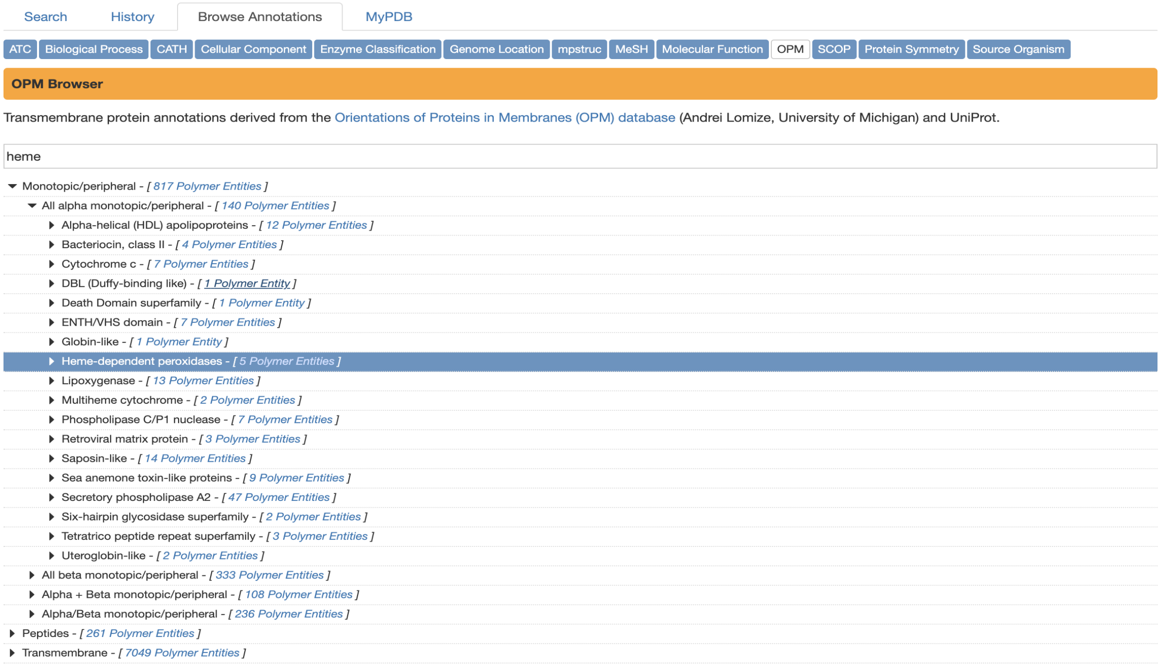


Tree browser, displaying OPM hierarchy. Enter a search term or navigate the tree. The link at the end of each line launches a search.

# Supplementary Figure 5

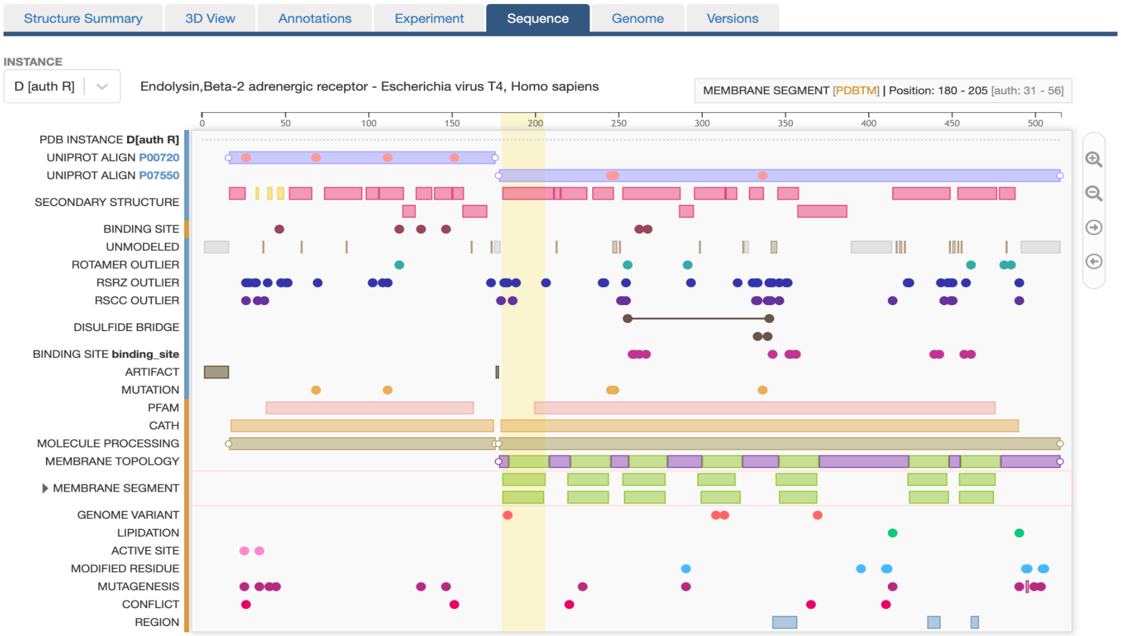


Membrane segment annotations from PDBTM and OPM associated with PDB ID 3SN6 chain D [auth R] in 1D Sequence View.
